# Feasibility of a Pediatric Acute Video Consultation Process Among Health Care Professionals in Primary Care in a Rural Setting: Protocol for a Prospective Validation Study

**DOI:** 10.2196/52946

**Published:** 2024-02-01

**Authors:** Marta Castillo-Rodenas, Josep Vidal-Alaball, Núria Solanas-Bacardit, Clotilde Farràs-Company, Aïna Fuster-Casanovas, Queralt Miró Catalina, Francesc López Seguí

**Affiliations:** 1 Centre d'Atenció Primària Cardona Gerència Territorial de la Catalunya Central Institut Català de la Salut Cardona Spain; 2 Cap de la Unitat de Suport a la Recerca de la Catalunya Central Fundació Institut Universitari per a la Recerca a l'Atenció Primària de Salut Jordi Gol i Gurina Sant Fruitós de Bages Spain; 3 Health Promotion in Rural Areas Research Group Gerència Territorial de la Catalunya Central Institut Català de la Salut Sant Fruitós de Bages Spain; 4 Faculty of Medicine University of Vic–Central University of Catalonia Vic Spain; 5 Direcció del Centre d'Atenció Primària Cardona Gerència Territorial de la Catalunya Central Institut Català de la Salut Cardona Spain; 6 Unitat de Suport a la Recerca de la Catalunya Central Fundació Institut Universitari per a la Recerca a l'Atenció Primària de Salut Jordi Gol i Gurina Sant Fruitós de Bages Spain; 7 Chair in ICT and Health Centre for Health and Social Care Research University of Vic–Central University of Catalonia Vic Spain

**Keywords:** primary health care, pediatrics, remote consultation, telemedicine, rural health services, video consultation

## Abstract

**Background:**

For years, in Catalonia and in the rest of Spain, there has been a deficit and an unequal geographical distribution of health professionals specializing in pediatrics, especially in rural areas. Among the proposals to improve this situation is the promotion of the use of information and communication technologies (ICT) among users and professionals. Moreover, with the outbreak of COVID-19, the use of telehealth has become an essential tool, with an overall increase in non–face-to-face visits, including in primary care pediatrics. In this context, telemedicine, when used in primary care pediatrics, can be an effective means of improving families’ access to medical care. Currently, in Catalonia, telemedicine involving patients and health professionals is used in pediatric primary care through telephone consultation and asynchronous teleconsultation (eConsulta). Video consultation is in practice not used, although it could have different applications.

**Objective:**

The aim of this study is to evaluate the feasibility of a video consultation process with physical examination in acute pediatric pathology in rural areas among primary care professionals. In addition, the level of satisfaction with these remote consultations will be assessed from the perspective of both the users and the health care professionals.

**Methods:**

We will conduct a prospective experimental study to analyze the possibility of using video consultation in pediatric acute care in primary care in central Catalonia (Spain). A minimum of 170 children aged between 0 and 14 years attending the primary care center (PCC) for acute illness for a period of 1 year will be included in the study. Initially, the telemetric visit, including a physical examination, will include a nurse at the patient and family’s side and a pediatrician who will participate remotely. Subsequently, the pediatrician will visit the patient in person and the physical examination and diagnosis made during the remote visit will be compared with the physical examination and diagnosis of the face-to-face visit, which is considered the gold standard.

**Results:**

Recruitment was planned to begin in the second half of 2023 and continue for at least 1 year. It is anticipated to be a good resource for a variety of acute pediatric conditions in primary care. The evaluation will focus on the feasibility of performing live remote visits and comparing their diagnostic accuracy with that of face-to-face visits.

**Conclusions:**

We believe that this study could provide evidence on the feasibility and diagnostic accuracy of video consultation in pediatric acute primary care in a rural setting, as well as on satisfaction with video consultations among both users and professionals. If proven useful in addressing the acute needs of children in a variety of situations, it could become a digital health tool that improves the overall pediatric primary care service in rural areas, for both families and professionals.

**International Registered Report Identifier (IRRID):**

PRR1-10.2196/52946

## Introduction

Over the last years, different demographic, social, and professional factors, such as the growth and depopulation of certain areas, changes in the pattern of use of services, and the lack of professionals with formal training, among others, have made it necessary to reshape pediatrics on a European scale [1]. Specifically in Catalonia (Spain), through the Strategic Plan for the Organization of Pediatric Care in Primary Care and the Health Plan of the Generalitat de Catalunya 2021-2025, several measures have been implemented, such as the development of pediatric territorial teams in some regions [2,3]. There are several difficulties in implementing these changes, particularly the lack of specialized professionals and their heterogeneous distribution, especially in rural areas.

In the human resources map for pediatric care in Catalonia, drawn up in 2018, there were 371 primary care teams with professionals specializing in pediatrics out of more than 400 primary care teams. It is believed that the situation has recently worsened. Of the total number of professionals, 28% worked in a coordinated and integrated manner according to the strategic plan. A survey conducted by the Catalan Society of Pediatrics in 2019 found that around 30% of pediatric positions in primary care were occupied by nonpediatric professionals, who do not need to be certified to provide pediatric care. In addition, the study found that 974 nurses were working in pediatric care without specific training. There are also clear differences in the coverage and availability of professionals between areas [4].

To improve this situation, the Catalan Society of Pediatrics proposes, in addition to increasing human resources and their distribution, the strengthening and optimization of information and communication technologies (ICTs) to facilitate the connection between users and professionals [4].

Likewise, with the emergence of COVID-19, digital health has become a tool commonly used in health care. The pandemic has revolutionized the care model of health systems worldwide and has redirected the system toward telemedicine, with a remarkable increase in remote visits, including in the pediatrics setting [5,6]. It has increased the use of tools such as telephone consultations and eConsulta, a type of asynchronous remote consultation integrated into the Catalan public health system [7-9]. Others, such as video consultation, have been more limited [10]. All of this has led to the question of whether digital tools can improve pediatric care, giving rise to the concept of telepediatrics.

Telepediatrics can be considered a subspecialty of telemedicine and can be defined as the use of ICT to provide health services to children at a distance [11]. In Catalonia, telepediatrics, in the form of telephone consultations and eConsulta, has become an integral part of health care, and in a trend similar to those in other countries, the COVID-19 pandemic has significantly altered the profile of pediatric primary care visit types. As a result, there is now a higher proportion of non–face-to-face visits than ever before [12]. A study indicates that, as of March 2020, in Catalonia, there was a drop of more than 80% in face-to-face pediatric visits compared to the previous year, along with 15 times more remote consultations. Subsequently, while the rate of face-to-face visits began to recover, it has not returned to pre–COVID-19 levels. More than 2 years after the pandemic, non–face-to-face visits continued to account for over 20% of the total [13].

Another type of virtual consultation in the Catalan health care system is the interconsultation, an asynchronous patient-free consultation between different health professionals, usually between primary care and specialized hospital care. It has been incorporated into daily clinical practice and validated by the Ethics Commission of the Barcelona College of Physicians [14]. The main types of this form of consultation are dermatological teleconsultation, teleaudiometry, the teleulcer program, and the tele-eyelid program [15]. Specifically, teledermatology has been established as a standard procedure to connect with the referral service in doubtful cases and before referring a patient face-to-face to specialized dermatologic care [16]. These types of services, moreover, have a positive environmental impact and show significant time savings for users [17].

In addition, at the Catalan Institute of Health (the main service provider of the Catalan health system), a pilot test is being carried out of the “digital briefcase,” which comprises a set of devices and accessories associated with a smartphone or tablet that allow for the provision of a basic level of health care; it aims to improve the capacity to resolve problems of primary care [18]. Some of the devices it incorporates are a blood pressure monitor with electrocardiographic rhythm, a portable complete electrocardiogram, a digital stethoscope, and a portable ultrasound scanner. It may incorporate other instruments that may be useful depending on the medical specialty.

Despite their potential to enhance communication by enabling visual contact, pediatric video consultations are not commonly used in the public health system [19]. This type of visit briefly emerged during the COVID-19 pandemic, but its current use is anecdotal [13]. It is well established that when video consultations are used by health care professionals, with one physically present alongside the patient, it improves the quality of the care process [20,21].

Video consultation has been applied in follow-up visits in pediatric hospital consultations for chronic illnesses such as diabetes, obesity, and mental health problems, as well as digestive, rheumatic, neurological, and even respiratory illnesses [22,23]. Also, in the hospital setting it has been used as a control after discharge and even to avoid days of hospitalization, with good results in terms of cost reduction and family satisfaction [24,25]. However, there are few studies on video consultation in pediatric primary care [26].

An additional possibility offered by video consultations, besides making a structured anamnesis, could be remotely carrying out physical examinations. This is especially important in cases of pediatric acute pathology, as children at these ages often have difficulties expressing their symptoms. Moreover, in pediatric cases, functional interdependence, that is, the functioning together of a child’s organs, can commonly cause an underlying pathology to lead to unclear or ambiguous symptoms. [27]. In response, simple medical devices have been made available for the general population. They are sold in packs and include a digital camera, otoscope, and stethoscope. Good examples of these are the devices sold by the brands TytoCare, approved by the US Food and Drug Administration (FDA), and HIGO, developed by the University of Warsaw [28,29]. These devices are used by caregivers when the child is ill. Clinical data are recorded and sent to the practitioner via a remote application and the practitioner responds with the appropriate diagnosis and treatment. This reduces physical presence in emergency services. Currently, these devices are used in some countries, mainly by private mutual insurance companies [30]. There are also similar devices, such as those of the Firefly brand, aimed at health care professionals [31]. However, in Spain there is little evidence of the use of similar devices in clinical practice. One of the few examples is Kidscare, which offers a telemedicine service aimed at schools [32].

There are several practical reasons to use teleconsultation with physical examination for acute pediatric consultations in a rural context. One is that in rural areas with distant clinics where a pediatrician is not available every day, the pediatric nurse could manage visits with remote support from a pediatrician in another center. Currently, telephone support is already provided in these cases. If this were done through video consultation, the support would be more comprehensive. The second reason is that during vacations or when the pediatrician in charge is on leave, it could be possible in certain cases to see the patient remotely. Finally, a clear benefit would be observed out of hours when a patient needs to be evaluated by a pediatrician.

In this context, the general objective of this protocol is to evaluate a video consultation process with physical examination for acute pediatric pathology in rural areas among primary care professionals. Specifically, the aim is to assess 3 elements: the feasibility of the process, its diagnostic accuracy compared to face-to-face consultation at the same time (considered to be the gold standard), and user and professional satisfaction.

To achieve these specific objectives, the study will analyze the technical and human possibilities of pediatric video consultation, the most appropriate reasons for consultation, the diagnostic accuracy compared to concurrent face-to-face consultation, the difference in duration between the 2 types of visits, the acceptance and satisfaction of patients and professionals using validated questionnaires, and incidents that may occur during virtual visits.

## Methods

### Study Design

#### Trial Design

This will be a prospective experimental study of an acute pediatric care process using video consultation with a physical examination among health professionals.

#### Patients, Scope, and Period of Study

The study will involve children aged 0 to 14 years attending PCC Cardona for acute health problems. The acute pediatric conditions selected for video consultation in this research will be identical to those encountered in regular consultations, including any condition that requires a medical visit. Commonly observed symptoms include fever, cough, runny nose, sore throat, earache, abdominal pain, vomiting, diarrhea, skin lesions, and similar conditions.

The Cardona PCC is part of the health region of central Catalonia. Located in a rural area, Cardona covers a total area of 143 km^2^ and serves an assigned population of nearly 5000 people. It comprises 5 family medicine and nursing teams, along with 1 pediatrics and pediatric nursing team, among other services.

Data collection will span at least 1 year to ensure the representation of all seasonal pathologies and was scheduled to commence in the second half of 2023. It is expected that by the end of 2024 all cases will have been collected and the data and conclusions can be analyzed. The results of the study will be published at the end of the project and will be presented in the form of a doctoral thesis.

#### Inclusion Criteria

The inclusion criteria will be patients aged 0 to 14 years who attend the Cardona PCC for acute conditions and who are authorized to participate by their legal representatives.

#### Exclusion Criteria

Exclusion criteria will be cases in which the legal representative does not allow participation, check-ups from the Healthy Childhood Programme, chronic illnesses and follow-up visits, consultations requiring immediate face-to-face medical assessment, and cases where there is a language barrier.

#### Sample Size Determination

The sample will be for convenience. Considering the main objective of the study (to analyze the diagnostic accuracy of telemedicine compared to concurrent face-to-face consultation), in order to estimate the required sample size, and given the absence of similar studies to predict this accuracy, it will be necessary to include 170 children. This sample size calculation is based on achieving a 95% confidence level, an 8% margin of error, and accounting for a replacement rate of 10%.

### Data Collection, Sources of Information, and Intervention

Children aged 0 to 14 years presenting to PCC Cardona with acute conditions will be selected using nonprobability convenience sampling. The procedure will be integrated into the daily pediatric clinical practice to facilitate recruitment. The decision to invite the patient and their family to participate in the study will be based solely on the daily workload of the health care professional (to allow time for both virtual and face-to-face visits).

The patient and their legal representative will be informed verbally and in writing of the purpose of the study and will be given the opportunity to choose whether or not to participate. If they agree, they will be asked to sign the informed consent form.

First, the patient will go to an office with the pediatric nurse and will connect via video call (Microsoft 365 Teams) with their regular pediatrician, who will conduct the anamnesis. Then, the nurse, under the guidance of the pediatrician, will perform the physical examination with approved digital devices, which will include a Firefly digital camera, a video otoscope, and a Littmann CORE digital stethoscope [31,33]. The Firefly camera and video otoscope are FDA-approved and hold certifications for FC, CE, RoSH, and ISO for medical devices. The stethoscope is FDA-approved and complies with Health Insurance Portability and Accountability Act standards [31,33].

To conduct the physical examination, a translated and adapted version of the diagnostic questionnaire for pediatric telemedicine created by Bittmann [34] during the COVID-19 pandemic will be used. The physical examination will be systematic. First, anamnesis will be conducted and the reason for the visit will be recorded. Subsequently, there will be a clinical examination. The final step will be the initiation of treatment.

The case identification data, date and time, age, gender, accompanying person, reason for the visit, and medical history, as well as the results of a physical examination (general appearance, skin, throat, otoscopy, cardiorespiratory auscultation, and other data, if necessary), diagnostic orientation, and duration of the visit will be recorded on the data collection sheet.

To ensure the effectiveness of digital health consultations, both professionals conducting the visit have received training in performing physical examinations using the devices intended for video consultations. Additionally, an incident registration section has been included to identify issues of any type (technical or human).

Afterward, the same pediatrician will again examine the child in person and record the physical examination with the apparatus. The diagnostic orientation and duration of the visit, as well as the concordance between the telemedicine examination and the diagnosis, will be evaluated in comparison to the face-to-face visit (considered the gold standard). In addition, in each case we will record whether the virtual visit was viable (when the anamnesis and physical examination were completed and a diagnosis was reached) and whether there were any incidents.

Validated questionnaires on satisfaction will be given to both the family and health care professionals (nurse or pediatrician) [35,36].

### Statistical Analysis

The aforementioned variables will be collected through Microsoft 365 Forms. For data analysis, R (version 4.0.3; R Foundation for Statistical Computing) will be used.

The accuracy of the video consultation diagnosis will be estimated by comparing it to the face-to-face diagnosis. The Pearson chi-square test will be used to analyze the relationship between pairs of categorical variables and the Student *t* test (2-tailed) or Mann-Whitney *U* test will be used to analyze the relationships between pairs of continuous variables. Categorical variables will be described as absolute frequencies and percentages, and continuous variables as mean and SD or median and IQR, depending on the distribution of each variable.

### Ethical Considerations

The study protocol, which involves human subjects, was reviewed and approved by the University Institute for Research in Primary Health Care Jordi Gol i Gurina (Barcelona, Spain) ethics committee (22/236-P). Written informed consent will be requested from all parents or legal guardians participating in the study. The original data collection will be conducted with informed consent that includes provision for secondary analysis of the data. The ethics committee has confirmed that the secondary analysis is covered under the initial consent procedure and does not require additional consent forms. Participants in this study will not be compensated.

To protect participant privacy and confidentiality, all study data will be anonymized and deidentified prior to analysis. The video consultation, conducted through the Teams platform, will not be recorded, and no images, sound, or personal data of any patient will be captured. In cases where a photograph is required, for example for teledermatology services, just as in a face-to-face visit, explicit consent will be requested from the patient and their family. Regarding the sounds of cardiorespiratory auscultations that might be recorded for transmission between the nurse and the pediatrician, the audio will not contain any user-identifiable data and will be deleted from the corresponding application at the end of the visit.

In this regard, the fundamental principles of the physician-patient relationship in telemedicine are the same as in a face-to-face visit. While telemedicine has a distinct impact on the physician-patient relationship framework, adherence to good clinical practice, and ethical and deontological standards, the legal and professional regulations applicable to any medical act must be followed [20]. In the practice of telemedicine during this study, special attention will be given to aspects of identification, trust, prudence, confidentiality, clinical information and communication, informed consent, and clinical judgment, as well as record-keeping of medical history and during treatment, follow-up, and evaluation.

## Results

Recruitment was scheduled to begin during the second half of 2023 and is expected to last 1 year and to include 170 patients. Preliminary results will be published by the end of 2024.

The visit via videoconference, with a physical examination of the patient performed by the pediatric nurse and the pediatrician attending remotely, is expected to be feasible and effective for most children with acute illnesses visiting primary care.

We believe that in other cases, such as when children are uncooperative or when the consultation is for specific reasons such as abdominal pain, video consultation may have limitations. In such cases, the reasons for this outcome will be recorded, and a face-to-face visit will always be conducted.

## Discussion

### Anticipated Findings

This study aims to provide evidence on the feasibility of, diagnostic accuracy of, and satisfaction with video consultation in the primary care pediatric acute care setting in a rural area. One of the traditional recommendations in telemedicine is not to use video consultations when the patient requires a physical examination, as is the case with acute pediatric pathology [21]. In this context, the purpose of this study is to test whether video consultation with a telemetric physical examination can be a viable and effective resource for acute pediatric conditions when there is on-site support from a health care professional. If video consultations are acceptable, they could partially alleviate the effects of the current shortage of pediatricians, particularly in rural areas.

Recommendations in telemedicine emphasize that non–face-to-face consultations should never be used as a way to enhance profitability during the workday or reduce the number of professionals [21]. In this study, these recommendations will not be breached; instead, there will be a redirection of the current approach used by pediatricians in conducting visits for acute conditions. This will ensure that children with acute conditions can be seen by a pediatrician without having to go to other urgent care centers or hospital emergency departments. Professionals must be aware that telemedicine cannot be a substitute for face-to-face examination of the patient when necessary. Therefore, telemedicine should be limited to cases where it is considered feasible. Setting limits on the use of telemedicine for patient care is a matter for the profession and professionals. Professionals must adhere to ethical and professional obligations with the same level of commitment as in face-to-face visits. Consequently, medical care must consistently maintain rigorous standards of both human and technical quality [20,21].

During the pandemic, the use of video consultation has provided an opportunity to understand its functionality and explore the potential it offers as a digital health tool in everyday medical practice. Regarding the potential benefits for pediatricians, just a few years ago it was difficult to imagine a virtual consultation that would involve examining a child, but the boost given to digital tools during the pandemic provides an opportunity to explore new modalities of care that can contribute to an optimization of resources while maintaining the quality of care and patient safety. In this context, it is important to train and raise awareness among professionals in order to determine when a visit can be carried out through a virtual consultation and when it is necessary to do it face to face [20,21]. Moreover, the study highlights the role of the pediatric nurse, who is physically present with the patient and family while the pediatrician guides the anamnesis and physical examination of the child from a distance. We believe involving pediatric nurses in this process will improve the quality of care and increase clinical safety [4].

It is also important to consider the views of health care professionals regarding telemedicine services. A recent study conducted in central Catalonia to evaluate the acceptance of telemedicine services found a positive reception, especially within the nursing community. Almost all participants agreed to continue using telemedicine in the future [37]. In relation to the benefits for health services, some articles have shown that telemedicine in general, and telepediatric health services for children living in rural areas in particular, can reduce the cost and travel time to access these services [38,39]. The use of teleconsultation can reduce the number of displacements, both for users and professionals, contributing to lower costs and time, increased efficiency, reduced pollution, and determining if there is overall greater satisfaction. On the scale of the public health system of Catalonia, studies have demonstrated that some of the digital tools that have been introduced, such as eConsulta, appear to be cost-effective. Others will need to be continually evaluated [40]. In turn, environmental benefits are gained by reducing the emission of atmospheric pollutants [41].

Video consultations can also be useful for health care providers. Health care organizations must develop practical strategies to consolidate the implementation of telemedicine and define new consultation structures to meet this new form of demand [20,21]. However, it should be noted that telemedicine in Spain does not have a specific legal regulation, as is the case in other countries in our context such as France, Sweden, Germany, or Switzerland. In the Spanish legal system and within the European framework, there are rules that are applicable and must be respected equally in both face-to-face and remote consultations [42,43]. Denmark and Israel, pioneers in the implementation of telemedicine, also have no specific legislation on the matter [44]. However, although the laws and regulations governing in-person medicine currently also apply to telemedicine, the development of specific laws and regulations will be essential.

Telemedicine has advantages and limitations in the human, technological, and economic spheres, and these must be understood by all parties involved. Remote consultations performed by health care professionals are medical procedures, and as such they are subject to current legislation based on the General Data Protection Regulation and the doctor-patient confidentiality relationship. For this reason, the use of safe and appropriate technological tools is essential [20].

### Limitations

This study has several limitations, primarily the risk of not achieving an accurate diagnosis through the virtual visit compared to the face-to-face visit. This could be due to technical or human causes (eg, if technological devices fail or if the child does not cooperate) or due to care-related factors related to, for example, abdominal pain, which requires careful palpation by an experienced professional to assess a possible acute abdomen. Other possible care demands not accessible at a distance may include eye and genital injuries, some wounds, and serious traumatic injuries. The fact that the video call is made between health care professionals, one of whom is always at the patient’s side, will improve the quality of care and reduce the risk of incidents. The presence of an assistant is a well-defined aspect of telemedicine [45,46]. What sets assistants apart is that they are health workers, which means that their role goes beyond merely relaying information; they actively participate in the visit. This collaborative approach is crucial for successful video consultations, where teamwork is essential. In this study, the assistant will be a pediatric nurse who will explain and accompany the families during the remote visit with guidance from the pediatrician. This guidance will include conducting anamnesis and physical examination, as well as providing information, diagnosis, and treatment to both the patient and parents.

Second, there may also be a patient selection bias, since depending on the patient’s characteristics (age, accompanying person, reason for consultation), some cases may not be selected if it is assumed that they are not suitable for virtual assistance. For this reason, an attempt will be made to carry out the study without the professionals knowing the characteristics of the patient or the reason for the consultation in advance.

Third, the fact that the same pediatrician performs both the telemetric and then the face-to-face visit may induce confirmation bias. Initially, the study was intended to be multicenter, with the participation of different professionals from different centers, but due to ethics and patient safety, as well as the lack of pediatricians, it was changed to a single-center intervention. Recording the physical examination and the diagnosis made during the virtual visit just prior to the face-to-face visit may mitigate this type of error.

Fourth, another possible limitation is nonacceptance by patients, family members, or the professionals themselves. There may be different reasons for this, including ethical, technological, and human reasons, as well as the risk of losing confidentiality. In this regard, detailed legislative regulation by the competent authorities of medical devices is very important. External validation of these devices must be essential to ensure efficacy and safety in real clinical practice and to be able to define the conditions under which they can be applied. Furthermore, providing adequate training to health care professionals and offering explanations to users is essential. As for the bioethical and patient safety aspects of virtual visits, currently, in Spain, they are comparable to those of face-to-face visits.

Last, other limitations may be the longer duration of virtual visits compared to face-to-face visits and the difficulty of conducting video consultations with families when there is a language barrier.

### Conclusion

We believe that this study can have significant future applications and implications, such as providing faster care to families, reducing time and travel for both users and professionals, mitigating the low ratio of pediatricians per population in certain rural areas, reorganizing territorial pediatric care, highlighting the role of the pediatric nurse as a trained professional capable of assessing pediatric needs, and optimizing resources for health service providers.

## Abbreviations

FDA: US Food and Drug Administration
ICT: information and communication technology
PCC: primary care center

## Data Availability

The data sets generated during and/or analyzed during this study are available from the corresponding author on reasonable request.
