# Correction: Feasibility of a Pediatric Acute Video Consultation Process Among Health Care Professionals in Primary Care in a Rural Setting: Protocol for a Prospective Validation Study

**DOI:** 10.2196/57937

**Published:** 2024-03-05

**Authors:** Marta Castillo-Rodenas, Josep Vidal-Alaball, Núria Solanas-Bacardit, Clotilde Farràs-Company, Aïna Fuster-Casanovas, Queralt Miró Catalina, Francesc López Seguí

**Affiliations:** 1 Centre d'Atenció Primària Cardona Gerència Territorial de la Catalunya Central Institut Català de la Salut Cardona Spain; 2 Cap de la Unitat de Suport a la Recerca de la Catalunya Central Fundació Institut Universitari per a la Recerca a l'Atenció Primària de Salut Jordi Gol i Gurina Sant Fruitós de Bages Spain; 3 Health Promotion in Rural Areas Research Group Gerència Territorial de la Catalunya Central Institut Català de la Salut Sant Fruitós de Bages Spain; 4 Faculty of Medicine University of Vic–Central University of Catalonia Vic Spain; 5 Direcció del Centre d'Atenció Primària Cardona Gerència Territorial de la Catalunya Central Institut Català de la Salut Cardona Spain; 6 Unitat de Suport a la Recerca de la Catalunya Central Fundació Institut Universitari per a la Recerca a l'Atenció Primària de Salut Jordi Gol i Gurina Sant Fruitós de Bages Spain; 7 Chair in ICT and Health Centre for Health and Social Care Research University of Vic–Central University of Catalonia Vic Spain

In “Feasibility of a Pediatric Acute Video Consultation Process Among Health Care Professionals in Primary Care in a Rural Setting: Protocol for a Prospective Validation Study” (JMIR Res Protoc 2024;13:e52946) the authors made one correction.

In the original manuscript, the affiliation of author Francesc López Seguí was listed as follows:

Centre for Health and Social Care Research, University of Vic–Central University of Catalonia, Vic, Spain

In the corrected version, it has been changed to read as:

Chair in ICT and Health, Centre for Health and Social Care Research, University of Vic–Central University of Catalonia, Vic, Spain

The correction will appear in the online version of the paper on the JMIR Publications website on March 5, 2024, together with the publication of this correction notice. Because this was made after submission to PubMed, PubMed Central, and other full-text repositories, the corrected article has also been resubmitted to those repositories.

